# A Comparative Evaluation of Carcass Quality, Nutritional Value, and Consumer Preference of* Oreochromis niloticus* from Two Impoundments with Different Pollution Levels in Zimbabwe

**DOI:** 10.1155/2018/7862971

**Published:** 2018-08-08

**Authors:** Vimbai R. Hamandishe, Petronella T. Saidi, Venancio E. Imbayarwo-Chikosi, Tamuka Nhiwatiwa

**Affiliations:** ^1^Department of Animal Science, Faculty of Agriculture, University of Zimbabwe, P. O. Box MP167, Mt Pleasant Harare, Zimbabwe; ^2^Department of Biological Sciences, Faculty of Science, University of Zimbabwe, P. O. Box MP167, Mt Pleasant Harare, Zimbabwe

## Abstract

The objective of the study was to determine the quality and consumer preferences of Nile tilapia (*Oreochromis niloticus*) from two water bodies with different pollution levels and trophic states. Water quality assessment of the two impoundments was carried out. Fish were sampled from hypereutrophic Lake Chivero and oligomesotrophic Lake Kariba for proximate analysis, carcass quality, and sensory evaluation. Conductivity, dissolved oxygen, transparency, ammonia, total phosphates, reactive phosphates, and chlorophyll a were significantly different (P<0.05). Fish from Lake Kariba had significantly higher condition factors and lower fillet yields, while fish of length 10-20 cm, from Lake Chivero had significantly more fat. Lake Chivero fish were darker, greener, and less red while Lake Kariba fish were lighter, less green, and less red. Raw fish from Lake Kariba were significantly firmer, were less green and redder, had a stronger typical fish odour, and were more acceptable than Lake Chivero fish. Lake Chivero fish had a stronger foreign fish odour than their counterparts. No statistical differences were observed on fillet cooking losses, cooked fish sensory parameters, and acceptability. The fish could, however, not be safe due to possibility of toxins in water and feed (algae) which may bioaccumulate and ultimately affect other attributes of fish quality.

## 1. Introduction

Fish meat quality is defined based on the sensory characteristics, chemical composition, and physical properties [1]. These quality attributes influence how the fish are perceived by the consumer [2]. Fish quality also involves safety aspects such as being free from harmful bacteria, parasites, biotoxins, pesticidal chemicals, heavy metals, and many other substances. According to FAO [3], wild fish in Zimbabwe are generally perceived to be of poor quality compared to aquaculture fish due to reasons such as off-flavours, spoilage, poor presentation, and linkage with polluted water bodies. Flos et al. [4] reported that the quality of fish is affected by exogenous and endogenous factors. Exogenous factors include diet composition, feeding frequency, and the fish environmental parameters such as salinity, pH, and temperature [2]. Endogenous factors are genetic and linked to the life stage, age, size, sex, and anatomical position in the fish [5]. In previous studies, differences in quality among Nile tilapia (*Oreochromis niloticus*) populations have been attributed to environmental factors [6, 7]. These factors influence body composition, sensory quality, and preferences in several fish species [7, 8].

Consumption of fish is associated with a number of benefits. As an important part of the diet, fish provides an affordable source of protein essential for human nutrition. More so considering that about 33 percent of Zimbabweans are undernourished, with 11 percent of children under the age of five moderately or severely underweight [3]. Because of their high protein content, polyunsaturated fats, vitamins, and minerals, fish can play a major role in alleviating malnutrition particularly in young children, pregnant women, and the elderly [9]. Fish consumption prevents cardiovascular diseases risk factors such as blood pressure, some types of cancer, Alzheimer's disease, and brain damage and does not increase obesity [10–12]. Despite the benefits of fish consumption, the* per capita *fish consumption in Zimbabwe, recorded at 3 kg/year in 2016, is low and below the SADC average (6.0 kg/year) [3, 13]. The Dietary Guidelines for Americans recommend consumption of 12 kg per year of seafood [14].

In Zimbabwe, Nile tilapia (*Oreochromis niloticus*) is the most common fish species and constitutes the dominant bream/tilapia caught by artisanal and commercial fishermen and is also produced intensively under aquaculture. It is now widely distributed in most reservoirs and river systems of Zimbabwe [3, 15]. Wild fish stocks occur in both Lake Kariba and Lake Chivero producing significant quantities of fish into the market. Lake Kariba is an oligomesotrophic water body, while Lake Chivero is hypereutrophic due to high levels of pollution with major sources of pollutants being sewage and industrial waste. These water bodies have different physical, chemical, and biological characteristics which influence the plankton, diversity of prey in the water bodies, and the substances to which the fish are exposed, thus offering different environments and nutrient sources.

Poor fish quality can be very detrimental to consumption, marketing, and acceptance of fish, yet fish is one of the best sources of animal protein [16]. Information should be available on the effect of different water quality status on fish attributes but is however scarce. This study hypothesized that the chemical composition, carcass quality, and sensory properties of fish from Lake Kariba and Lake Chivero are different due to the differences in water quality caused by extensive water pollution in Lake Chivero. In this study, this hypothesis of the link between fish quality and source water quality (exogenous factors) is tested. With the growing demand for fish and fish products, it is essential to determine the influence of water quality on fish since environment is considered the main factor controlling wild fish quality, its growth, and production [17]. This study was conducted to investigate carcass quality, nutritional properties, and acceptability of raw and cooked fish,* O. niloticus, *reared in impoundments with different trophic states.

## 2. Materials and Methods

### 2.1. Study Area

The study was carried out at the University of Zimbabwe, Department of Animal Science, and University Lake Kariba Research Station. Fish samples (*Oreochromis niloticus*) from Lake Chivero and Lake Kariba were used in this study. Lake Chivero is situated on the Manyame River and is the fourth largest impoundment in Zimbabwe. It is located 37 km southwest of the capital city of Zimbabwe, Harare, at latitude 17° 54′ S and longitude 30° 48′ E [18]. All major rivers such as Manyame, Mukuvisi, and Marimba and tributaries that discharge into Lake Chivero pass through the city of Harare and industrial sites, causing heavy water pollution [19]. The heavy pollution has resulted in the lake being classified as hypereutrophic. The lake stratifies in summer and overturns in the beginning of the winter season of each year [20]. The lake is loaded with nutrients especially in winter and there is high growth of phytoplankton due to nutrient availability [21].

Lake Kariba, located at 16.5°S and 28.8°E with an altitude of 518 m above sea level, is the largest inland dam in Southern Africa. It is a warm oligomesotrophic lake with three distinguishable seasons which are a hot rainy season (November-March), cool dry season (May-August), and a very hot dry season (September-November) [22]. The lake is divided into five geographical and limnologically distinct basins, namely, Mlibizi (basin 1), Binga (basin 2), Sengwa (basin 3), Bumi (basin 4), and Sanyati (basin 5) [23]. The levels of pollution differ in these basins with Sanyati basin receiving runoff water from farms, sewage, and mining drainage effluent from Kwekwe through Sebakwe River [24, 25].

### 2.2. General Water Quality Parameters

Water samples were collected from Lake Chivero and Lake Kariba from five sites for each lake at the same time as fish sampling. Sampling was carried out in June and October. Water samples were collected using a Ruttner sampler mounted to a speed boat. Vertically integrated samples were collected into 500 ml sterile bottles and immediately placed on ice in cooler boxes. Water quality parameters, namely, pH, conductivity, dissolved oxygen, and percent oxygen, were measured using handheld pH/oxygen/conductivity meters equipped with a cellOx 325 Oxygen Sensor (WTW), a SenTix 20 probe (WTW), and a conductivity sensor (WTW). Analysis was done on filtered samples to measure total nitrogen, total phosphorus, ammonia nitrogen, nitrate nitrogen (NO3-N), and orthophosphate phosphorus (PO4-P) according to Bartram and Ballance [26].

### 2.3. Fish Samples

Fish samples (*Oreochromis niloticus*) were obtained from the two lakes using seine nets. Sampling was done in June and October to determine the effect of season on fish nutritional composition, sensory characteristics, and carcass quality. Fish were also bought from local fishermen to get the required numbers and sizes. The fish were immediately transferred to the coolers and kept on ice until they were collected for further analyses. The fish were filleted and half of the fillets, approximately 50 grams, were used for individual measurement of cooking loss, protein, water, and fat content, and colour determination. The skin and all visible parts of fat were removed prior to analysis. All the fishes were individually analysed. The other fillets were used for sensory evaluation. These fillets were wrapped in plastic bags and frozen at −20°C.

### 2.4. Biometric Traits

Biometric parameters were measured before degutting, descaling, skinning, beheading, and harvesting of fillets using standard methods. These were total length (TL) in centimeters (cm), total weight (TW), dressed weight (DW), liver weight (LW), and fillet weight (FW) in grams (g). Dissection was done using a sharp knife and scissors.

The following parameters were calculated:

(1) Condition  factorCF=fishTWTL  x  TL  x  TL×100Hepatosomatic  indexHSI%=100×LWgTWgDressing  indexDW%=100×DWgTWgFillet  weightFW%=100×FWgTWg

### 2.5. Nutritional Quality of Fish

A total of forty fish fillet samples were subjected to moisture, ash, fat, and protein analysis using standard methods which are detailed in Association of the Official Analytical Chemists [27].

### 2.6. Percentage Cooking Loss

A total of forty frozen fish fillets were thawed at 3°C for 12 hours. Using a Faizco SF-820 laboratory digital scale, 100 g was taken from each fillet for determination of cooking loss. Each fillet was placed in tight Polyvinyl chloride (PVC) bags and cooked in a water bath heated at 100°C for 20 minutes and then cooled to room temperature. Water released after cooking and cooling was manually separated by pouring out the water from the PVC bag and the weight of the fillet was taken.

The cooking loss was calculated using the following formula:

(2) Cooking  loss  %=100×weight  of  cooked  fish  muscletotal  muscle  weight

### 2.7. Colour Determination

The fish fillets were analysed for colour by first thawing at 4°C for 4 hours. A high resolution digital Cannon Powershot SX400IS, with 16 megapixels, was used to take pictures of the fish fillets. The camera was secured on a tripod and lens faced downward towards the fillet sample. Images of the fillets were taken using day light conditions and uniform light intensity. The images were saved on a memory card and transferred to the computer where Adobe Photoshop extended CS6 was used as described by Yam and Papadakis [28]. The histogram window was used to determine the colour distributions along the x-axis and y-axis on the fish fillets. Five points, measuring 1.2 × 1.4 inches, were cropped on each fillet using Photoshop in order to obtain the L, a, and b values. The window displayed the statistics (mean, standard deviation, median, percentage, etc.) of the colour value, lightness (L) with lightness=0 for black and lightness = 100 for white, redness (a), and yellowness (b), for a selected area on the fillet image. The figures obtained were converted to L*∗*, a*∗*, and b*∗* since the histogram values are not standard colour values.

The following formulae were used:

(3) L∗=Lightness225×100a∗=240a255−120b∗=240b255−120

### 2.8. Evaluation of Raw Fish

Thirty whole raw fish from Lake Kariba and from Lake Chivero with mean weight ranging from 350 g to 450 g were evaluated by 15 trained panelists from the Faculty of Science and Faculty of Agriculture. The fish skin, belly flaps, peritoneal area, fillet, and general characteristics were evaluated in terms of colour (greenness, blackness, and redness), texture (firmness), visible fat deposition, odour (fresh fish odour intensity, foreign odour intensity), and acceptability. The parameters were ranked using a five-point hedonic scale with descriptors for each fish part and parameter. Prior to the assessment, frozen samples from each source were thawed overnight at 4°C. Whole fresh fish samples from each source were placed on trays and simultaneously presented to the panelists.

### 2.9. Consumer Preference

A consumer preference test was performed on fish samples from the two lakes, Kariba and Chivero, using the method of Stone and Sidel [29] and Resurrecion [30]. A panel of 154 untrained individuals drawn from college students who ate fish was used. Frozen fish fillets were defrosted for two hours at room temperature and the upper portion was cut into four individual portions weighing 50 grams each. Sixty portions from each source were then prepared by steam cooking in plastic cooking bags for twenty minutes and boiling temperature. No flavouring or spices were added to the fish fillets. The panelists indicated their preferences based on four sensory attributes, namely, taste, colour, flavour, and texture on a hedonic scale of one to five based on like/dislike. Sensory judgments were scored as follows: 1: dislike extremely, 2: dislike moderately, 3: neither like nor dislike, 4: like moderately, and 5: like extremely. The panelists rinsed their mouths with water before and after each sample.

### 2.10. Data Analyses

The Statistical Analysis System (SAS) version 9.3 (SAS, 2010) was used to analyse data on carcass quality, nutritional composition, and sensory evaluation. The Shapiro-Wilk test was used to determine if all sample data had been drawn from normally distributed populations using the PROC UNIVARIATE PLOT NORMAL procedure of SAS. All data on water quality, carcass, and nutritional composition parameters were analysed using the PROC GLM of SAS using the following model:

(4) $$\begin{array}{c} y_{ijk}=\mu +L_{i}+S_{j}+{\left(\;LS\;\right)}_{ij}+e_{ijk} \end{array}$$

where** y**_**ijk**_ were the observed water quality, nutritional composition, and carcass parameters and** L**_**i**_,** S**_**j**_, and** (LS)**_**ij**_ were the fixed effects of the source, season, and interaction between source and season, respectively. The ***μ*** and** e**_**ijk**_ were the overall mean and random residuals, respectively. Means were separated using the adjusted Tukey's method. Differences were considered statistically significant at* P<*0.05. The Kruskal-Wallis test was used to determine the effect of source of fish and month of sampling on colour, texture, visible fat deposition, typical fish odour, and foreign odour with the PROC NPAR1WAY analysis of data. Sensory evaluation data were obtained as scores collected on a 5-point hedonic scale. The data were therefore not drawn from a normally distributed population as was confirmed by the Shapiro-Wilk test. Mean scores from the sensory evaluation of raw and cooked fish were presented using spider web plots. Hedonic scores for each of the organoleptic variables on fish from Lake Kariba and Lake Chivero were compared with the t-test Approximation in a Wilcoxon two-sample test derived from a PROC NPAR1WAY analysis of SAS. A principal component analysis was carried out with PROC PRINCOIMP of SAS to identify the major components influencing consumer preference for fish from Lake Chivero and Lake Kariba. The most important principal components were determined from the magnitude of the eigenvalues for Lake Chivero and Lake Kariba as well as cumulative proportion. 

## 3. Results

### 3.1. Water Quality of Lake Kariba and Lake Chivero

Water quality results of the two lakes are shown for the months of June and October (Table 1). Conductivity, oxygen percent, temperature, ammonium, reactive phosphorus, and chlorophyll a were statistically different between the two lakes and were influenced by month, lake, and their interaction (*P<*0.05). Total phosphates, nitrates, and transparency were influenced by source only while pH was influenced by month (*P<*0.05). The two water bodies showed significant differences in water quality.

### 3.2. Carcass Quality

Carcass quality results are shown in Table 2. Fish from Lake Kariba had significantly higher condition factors (*P<*0.05) in both the months of June and October than fish from Lake Chivero. Fish from both lakes had lower condition factors in the month of June than in October. Fillet yield was significantly higher for fish from Lake Chivero than fish from Lake Kariba in the month of October (*P<*0.05). There were no significant differences in fish hepatosomatic indices.

### 3.3. Nutritional Composition

Data in Table 3 shows nutritional composition of fillets from fish of medium size, 10-20cm. Source and month of fish sampling had no significant effect (*P<*0.05) on protein, ash, and DM while the interaction between source and size of the fish influenced fat content. Fat content was significantly higher (*P<*0.05) in fish sampled in the month of October from Lake Chivero for the medium sized fish. There were no significant differences observed in fish of length >20 cm between the two water bodies.

### 3.4. Percentage Cooking Loss

There were no differences in cooking loss between fish fillets from the two lakes across the experimental months (*P>*0.05). Least square means for cooking losses for fillets from Lake Kariba and Lake Chivero were 20.74% and 20.76%, respectively.

### 3.5. Fillet Colour

The measured colour of* Oreochromis niloticus* samples evaluated in this study reflected variability in terms of lightness, a*∗* and b*∗* (*P<*0.05) (Table 4). Fish fillets from Lake Chivero were significantly darker (lower lightness) (*P<*0.05) than those from Lake Kariba for both months, just as fillets were lighter in October than in June in both lakes (*P<*0.05). Fish fillets from Lake Chivero were greener and less red than those from Lake Kariba and the latter were redder and less green as shown by the chromatic component a*∗* values (ranges from -120 to 120, from green to red). There were no differences in chromatic component b*∗* (ranging from -120 to 120, blue to yellow) between fish fillets from the two lakes. However, fish fillets from Lake Chivero had more yellowness in October than in June (*P<*0.05).

### 3.6. Evaluation of Raw Fish

**Figure 1:**
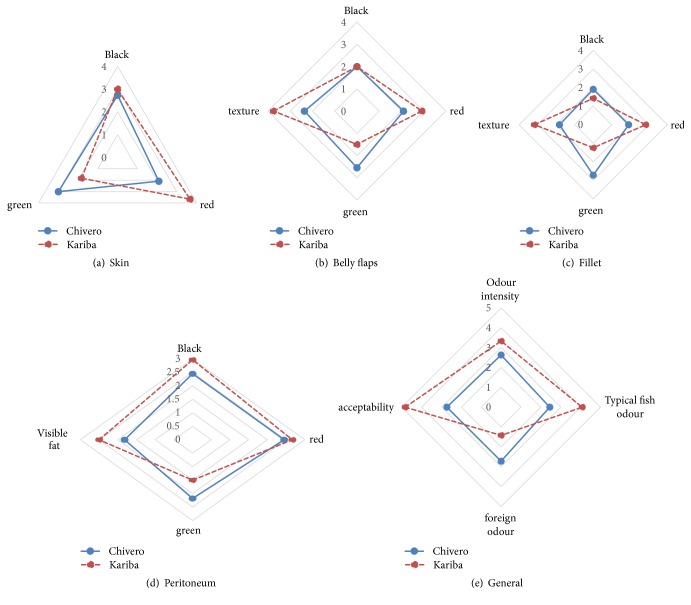
Comparison of raw Nile tilapia from Lake Kariba and Lake Chivero. (a) Colour of fish skin. (b) Colour and texture of belly flaps. (c) Appearance of peritoneum. (d) Colour and texture of fillets. (e) General odour and acceptability of the fish.

Figure 1 shows the mean scores for the raw fish parameters. The skins of fish from Lake Kariba were significantly more red than skins from Lake Chivero (*P<*0.05) while Lake Chivero fish fillets and belly flaps were significantly more green (*P<*0.05) than Lake Kariba fillets. The texture of fish from Lake Kariba was perceived to be significantly firmer (*P<*0.05) than that of fish from Lake Chivero as these were scored higher than Lake Chivero. In terms of the typical fish odour, Lake Kariba fish had a significantly stronger (*P<*0.05) typical fish odour than fish from Lake Chivero. Foreign odour was significantly stronger in fish from Lake Chivero than the counterparts. Acceptability of fish from Lake Kariba was significantly higher (*P<*0.05) than that of fish from Lake Chivero.

### 3.7. Consumer Preference and Effect of Source

**Figure 2:**
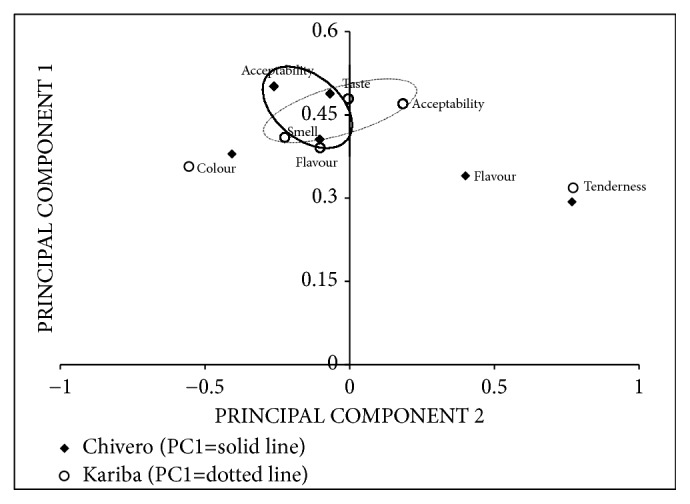
Component patterns for PC1 and PC2 for fish from Lake Kariba and Lake Chivero.

**Figure 3:**
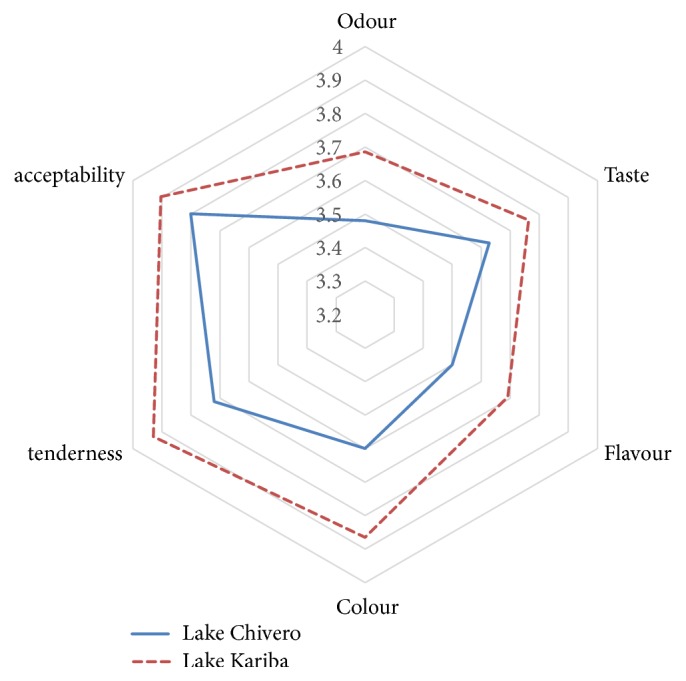
Comparison of sensory parameters for cooked fish from Lake Kariba and Lake Chivero.

The major components that influenced consumer preference are shown in Table 5. The eigenvalues show that PC1 was the most important component for fish from both Lake Chivero and Lake Kariba. Although PC2 had loadings below one, it was also relatively important since when combined with PC1, they contributed the majority of the variability that was observed among the six variables (smell, taste, acceptability, tenderness, flavour, and colour). As such, only PC1 and PC2 were retained. These contributed a cumulative total of 62.2% and 61.6% of observed variability among the six organoleptic variables in Lake Kariba and Lake Chivero, respectively. PC1 comprised smell, taste, and acceptability for both lakes (Figure 2). All other parameters had little individual significance, if any, as far as influencing consumer preference was concerned. Results from t-test approximation of the two-tailed Wilcox two-sample test indicated that there were no significant differences (*P>*0.05) in organoleptic scores for smell, taste, flavour, colour, tenderness, and acceptability between cooked fish sourced from Lake Kariba and Lake Chivero (Figure 3).

## 4. Discussion

Water quality results obtained in this study reflected the differences that exist between Lake Kariba and Lake Chivero. Differences in conductivity, dissolved oxygen, oxygen percent, temperature, ammonium, reactive phosphorus, and chlorophyll a were due to the extensive pollution in Lake Chivero. The pollution is caused by sewer and industrial effluents from neighboring city of Harare and industrial sites depositing raw effluent into the lake [31, 32]. High levels of degradable material are more oxygen demanding and this depletes dissolved oxygen in the water body [33]. The high level of nutrients, especially phosphorus, supports growth of different phytoplankton and macrophyte species including water hyacinth which deplete the water body of dissolved oxygen, thus the low oxygen levels in Lake Chivero compared to Lake Kariba [34, 35]. Dissolved oxygen is one of the major factors that affect fish growth [22].

Fish from Lake Kariba had a higher condition factor than from Lake Chivero probably due to the differences in water quality and type of food available in the two water bodies. Higher condition factors reflect a better nutritional status and better adaptation of the fish to its immediate environment [36]. However, the condition factor of fish from Lake Chivero was expected to be higher than that of fish from Lake Kariba to relate well with the high nutrient state which supports growth of phytoplankton, a source of food for the fish [21]. The observed poor condition of the fish might have been attributed to the stress resulting from pollution. Highly polluted water resources such as Lake Chivero are characterized by conditions that negatively affect fish development and proper functioning of internal organs such as the low dissolved oxygen [6]. In a study by Khallaf, Gala, and Authman [37], the level of pollution and season were the most important factors that influenced fish condition factor. Similar condition factors of 1.9-2.4 were reported by Asaminew, Tefera, and Tadesse [38] on* Oreochromis niloticus* from different water sources and Utete et al. [22] on the same species from Lake Chivero. Seasonal effects were also observed to influence fish condition factor. In the month of June, fish had lower condition factors than those sampled in October probably due to differences in water temperature. Water temperature ranging from 28°C to 32°C is optimum for survival, growth, and reproduction of* Oreochromis niloticus* [39]. In the month of June water temperatures were below the average optimal growth temperatures for fish as they averaged 16.5°C in Lake Chivero and 24°C in Lake Kariba. Condition factors were higher in the month of October possibly due to higher temperatures close to the optimal temperature for fish growth and survival.

Fish from Lake Chivero had a significantly higher fillet yield than fish from Lake Kariba in October. The variability of fillet weight while dressing-out percentage was not different could be attributed to differences in diet, fish age, sex, season of capture, and environmental conditions and to some extent techniques of filleting [40]. A higher fillet percentage from the fish is desirable since it leads to a higher yield of edible portions and subsequent reduction in the quantity of processing waste [41].

Muscle composition of fish flesh has a lot of influence on how the fish is generally perceived by the consumer in terms of its taste, flavour, and general acceptability [40]. The differences noted on ether extract and dry matter between fish from Lake Kariba and Lake Chivero are not surprising as nutritional value of most freshwater fish has been reported to differ between water bodies, fish size, and season [42]. Lake Chivero is highly polluted and so the feed resources and stress factors for fish in this lake are different when compared to Lake Kariba. Fish in the length class 10-20 cm from Lake Chivero had the highest fat content and the least was from Lake Kariba in the same month. Normal fat content for* Oreochromis niloticus *is 2.75±0.16% but however varies with the geographical region, diet, season, sexual maturity, and age [42]. Considering the fat content of all the fish samples, all the fish were found to be classified as lean. Lean fish have their fat content less than 5%.

The values of DM and ash obtained in this study compared well with the DM and ash of wild* Oreochromis niloticus* reported by Olopade, Taiwo, Lamidi, and Awonaike [43]. Protein content for all the fish from the two lakes in the two seasons was within the range that is classified as high which is greater than 15% [42]. When taking into account only the nutritional requirements for human health, fish from both sources can be reliable sources of nutrients if included in the diet but the fish may not be safe due to possibility of toxins and other poisonous substances that might accumulate in fish which include heavy metals and toxins.

Colour is an important fish quality parameter that has a significant influence on consumer acceptance and also its market value since consumers associate fish flesh clarity with product freshness [44]. The evaluation of fish fillets showed colour differentiation between fish from Lake Chivero and from Lake Kariba. The greenness and dark colour in fish fillets from Lake Chivero can probably be attributed to presence of pigments that are produced by phytoplankton and macrophytes, differences in fat content, water transparency, and higher deposition of melanin due to dietary effects [2, 45, 46]. These results are in agreement with the differences noted in the assessment of raw fish. Colour differences in cichlid species have been attributed to environmental variations which include water transparency [46]. Kop and Durmaz [45] indicated that there were pigments produced by phytoplankton and plants, namely, melanine, purines, pteridiums, and carotenoids which give colour to the skin and flesh of many fish species. The differences in water quality, therefore differences in phytoplankton species and biomass between Lake Chivero and Lake Kariba, could explain the differences in colour between the fish. Some authors reported a direct relationship between fat content and whiter fillets which was not consistent with the observations in this study [47].

Important differences were also noted on raw* Oreochromis niloticus* from Lake Chivero and Lake Kariba. Assessors perceived Lake Kariba fish to be significantly firmer than fish from Lake Chivero. Textural differences observed with fish from Lake Kariba being firmer can be due to differences in fat content, nutritional state of the fish, water content, and activity [48, 49]. Fish from Lake Chivero had more fat and more moisture than fish from Lake Kariba, which could have contributed to the differences in texture. The stronger foreign odour intensity of fish from Lake Chivero could have been due to compounds found in the fish environment. Some of the compounds include those that are produced by blue-green algae and actinomycetes called geosmin and 2-methylisoborneol [50]. These compounds have strong odour characteristics that influence the odour of fish. Differences in phytoplankton communities in the water bodies may explain the differences in fish odour [51].

In the consumer preference study, cooked fish was considered to have similar organoleptic characteristics, independent of origin. Although the raw fish had different properties such as texture, odour, and colour, these differences were not detected on cooked fish in the consumer preference study. Cooking could have altered some of the properties since cooking especially by boiling and steam cooking alters organoleptic properties of fish muscles [52]. The results indicate that some perceptions that consumers have on quality of fish can be a result of consumer beliefs and physical characteristics of the raw product.

## 5. Conclusions

The study has demonstrated the differences in fish meat quality that arise due to pollution of water bodies. The information generated can be used to determine the suitability of some ecosystems to produce fish that meet important fish meat quality specifications. Lake Kariba offered a better and adaptable environment for fish condition although fish from Lake Chivero produced higher fillet yields. Fillet colour was different as Lake Chivero fish were darker and greener. Despite the differences in fish environment, fish from both sources met human protein requirement. Typical fish odour was stronger in fish from Lake Kariba than fish from Lake Chivero while foreign fish odour was stronger in fish from Lake Chivero than fish from Lake Kariba. Acceptability of raw fish was higher for fish from Lake Kariba than from Lake Chivero while cooked fish from both sources were acceptable. However, the presence of toxins and microorganisms in water due to pollution will ultimately affect other attributes of quality. The impact of source on levels of contaminants such as heavy metals and biotoxins and microbiological status of fish needs to be investigated.

## Figures and Tables

**Table 1:** Least square means for water physicochemical parameters for Lake Chivero and Lake Kariba in June and October.

**Parameter**	**Units**	**Lake Chivero**		**Lake Kariba**		**s.e.**
		**June**	**October**	**June**	**October**	
pH		7.67	8.28	8.02	8.44	0.230
Conductivity	*µ*S/cm	513.00^a^	683.60^a^	93.36^b^	71.26^b^	42.540
Oxygen percent	%	30.68^a^	112.00^b^	99.12^b^	90.54^b^	5.992
Dissolved oxygen	mg/l	2.57^a^	7.98^b^	7.93^b^	6.72^b^	0.381
Temperature	°C	16.40^a^	25.38^a^	24.28^b^	27.58^b^	0.467
Transparency	m	0.86^a^	0.64^a^	2.40^b^	1.94^b^	0.230
Ammonia	mg/l	998.36^a^	216.74^b^	50.40^b^	29.70^b^	156.761
Total phosphorus	mg/l	804.56^a^	833.44^a^	32.20^b^	24.24^b^	77.60
Nitrates	mg/l	248.24	111.94	37.72	32.22	53.636
Total nitrogen	mg/l	1246.64	1548.62	85.32	2023.84	816.739
Reactive phosphorus	mg/l	739.22^a^	543.58^b^	13.84^c^	11.66^c^	40.167
Chlorophyll A	*µ*g/l	3.70^a^	12.92^b^	1.14^ac^	2.22^ac^	1.420

Least squares means with similar superscripts within rows are nonsignificantly different (P>0.05).

**Table 2:** Carcass quality of *Oreochromis niloticus* from Lake Kariba and Lake Chivero.

	**Lake Chivero**		**Lake Kariba**		
**Variable **%	**June**	**October**	**June**	**October**	**s.e.**
Condition factor	1.86^a^	2.02^ab^	2.12^bc^	2.24^cd^	0.047
Hepatosomatic index	0.96	1.23	1.03	0.76	0.112
Dressing index	89.40^a^	88.30^ac^	86.72^ad^	84.83^cd^	0.971
Fillet yield	38.26^ab^	38.92^a^	35.58^b^	32.00^c^	0.858

Least square means with similar superscripts within rows are not significantly different (p>0.05).

**Table 3:** Least squares means for nutritional composition (%) of fish of size 10-20 cm of total length from Lake Chivero and Lake Kariba.

Source	Season/month	EE	CP	Ash	DM
Chivero	June	1.86^b^	18.20	1.15	20.06^a^
	October	3.14^a^	19.69	1.62	19.39^ab^

Kariba	June	1.60^b^	17.93	0.90	19.55^ab^
	October	0.83^bc^	18.32	0.81	18.45^b^

	s.e.	0.274	0.435	0.278	0.272

LS means with similar superscripts across columns are nonsignificantly different (p>0.05) for the principal components.

**Table 4:** Least square means for lightness and chromatic components (s.e.).

**Colour parameter**	**Lake Chivero**		**Lake Kariba**	
	**June**	**October**	**June**	**October**
Lightness	49.00(1.077)^d^	54.68(1.077)^b^	60.83(1.077)^a^	51.45(1.115)^c^
a*∗*	-0.08(0.445)^c^	4.17(0.443)^a^	2.42(0.443)^b^	2.25(0.458)^b^
b*∗*	10.09(0.406)^d^	11.79(0.406)^bc^	12.70(0.406)^ac^	13.54(0.420)^a^

**Table 5:** Eigenvalues for the principal components of fish from Lake Chivero and Lake Kariba.

Principal component	Lake Kariba (1)			Lake Chivero (2)		
	Eigenvalue	Proportion	Cumulative proportion	Eigenvalue	Proportion	Cumulative proportion
**1**	**2.810**	**0.468**	**0.468**	**2.802**	**0.467**	**0.467**

**2**	**0.926**	**0.154**	**0.622**	**0.895**	**0.149**	**0.616**

3	0.788	0.131	0.753	0.764	0.127	0.743

4	0.592	0.099	0.852	0.727	0.121	0.864

5	0.448	0.075	0.927	0.503	0.084	0.948

6	0.436	0.073	1.000	0.309	0.052	1.000

## Data Availability

The data used to support the findings of this study are available from the corresponding author upon request.
